# Supplementary material for: Clinical and dosimetric correlation in terms of treatment response, bladder and rectal toxicities in cervical cancer patients treated with cobalt 60 high dose rate brachytherapy
Source: PeerJ. 2024 Aug 22;12:e17759. doi: 10.7717/peerj.17759 (PMC11345003; doi:10.7717/peerj.17759)
Supplement: Supplemental Information 2 [file peerj-12-17759-s002.docx]

STROBE Statement—Checklist of items that should be included in reports of ***cross-sectional studies***

|  | Item No | Recommendation |
| --- | --- | --- |
| **Title and abstract** | 1 | (*a*) Indicate the study’s design with a commonly used term in the title or the abstract  The study design has been mentioned in the methods section in the manuscript  Page 3 , line 56  “A longitudinal descriptive study was conducted at Kasturba Medical College Hospital, Attavar, Mangalore between..” |
|  |  |  |
|  |  | (*b*) Provide in the abstract an informative and balanced summary of what was done and what was found.  Page 1  These information are stated in the study abstract (study objective described, method and results described) |
| Introduction | | |
| Background/rationale | 2 | Explain the scientific background and rationale for the investigation being reported  Page 2  Rationale and existing literature are stated in the introduction section |
|  |  |  |
| Objectives | 3 | State specific objectives, including any prespecified hypotheses  Page 2  A statement at the end of the introduction specifies the specific goals and objectives.  The aim of our study is to evaluate clinical and dosimetric parameters in terms of tumour response, bladder, and rectal toxicity in patients undergoing Cobalt 60 HDR Brachytherapy. |
| Methods | | |
| Study design | 4 | Present key elements of study design early in the paper  Page 3, line 56  Study design is stated in the first subsection of Methods. Key elements are all described in the methods  “A longitudinal descriptive study was conducted at Kasturba Medical College Hospital, Attavar, Mangalore between September 2020 to June 2022 after obtaining the institutional ethics approval ( Kasturba Medical College, Mangalore , Manipal academy of higher education,Deemed to be university granted ethical approval to carry out the study within its facilities with Protocol No. IEC KMC MLR 12/2022/420) “ |
| Setting | 5 | Describe the setting, locations, and relevant dates, including periods of recruitment, exposure, follow-up, and data collection  Setting, locations, and relevant dates, including periods of recruitment, exposure, follow-up, and data collection are described in the methods and material sections  Page 3 and 4  “CT scan simulation was done with applicators in-situ with 50ml of diluted contrast in bladder without intravenous contrast. CT 3mm thickness axial sections were taken. The CT files were then transferred to SAGI TPS. Applicators and catheters were reconstructed in the brachytherapy planning module (Figure 1). We prescribed the High-risk clinical target volume (HR CTV) and contours the organs at risk (OAR) such as bladder and rectum as per Gyn GEC ESTRO contouring guidelines. Inverse planning technique was followed. Further optimization was done manually by dragging isodose line using graphical tool. This was a trial and error process where dwell time/dwell position are adjusted to achieve optimal dose coverage to High risk clinical target volume (HRCTV) (EQD2>80Gy10) with constraints to bladder D2cc (EQD2<85Gy3) and rectum D2cc (EQD2< 75 Gy3)(Viswanathan et al., 2009).” |
| Participants | 6 | Give the eligibility criteria, and the sources and methods of selection of participants  Study population is described is the method section  Page 3  “Patients who met the eligibility criteria of histopathologically proven carcinoma cervix FIGO stage 1B to IVA , with their written informed consent to participate were provided a questionnaire with demographic, basic medical information. According to the proforma, retrospective analysis parameters of the patients was extracted from medical records department and for prospective analysis data was collected on case to case basis. Dosimetric parameters was extracted from SAGI plan”. |
| Variables | 7 | Clearly define all outcomes, exposures, predictors, potential confounders, and effect modifiers. Give diagnostic criteria, if applicable  Standardized variable definitions were used across all programs, which are presented in method section.  Page 3  “Clinical response was assessed at 3 months after brachytherapy completion. Response was evaluated with MRI in accordance with RECIST criteria 1.1. Bladder and rectal acute toxicities were assessed at end of 3 months. Acute complications were assessed according to RTOG acute toxicity criteria at end of 3 months(Cox et al., 1995).” |
| Data sources/ measurement | 8* | For each variable of interest, give sources of data and details of methods of assessment (measurement). Describe comparability of assessment methods if there is more than one group  Data collection and measurement was the same for all variables, and is described in the methods section.  Page 3  “CT scan simulation was done with applicators in-situ with 50ml of diluted contrast in bladder without intravenous contrast. CT 3mm thickness axial sections were taken. The CT files were then transferred to SAGI TPS. Applicators and catheters were reconstructed in the brachytherapy planning module (Figure 1). We prescribed the High-risk clinical target volume (HR CTV) and contours the organs at risk (OAR) such as bladder and rectum as per Gyn GEC ESTRO contouring guidelines. Inverse planning technique was followed. Further optimization was done manually by dragging isodose line using graphical tool. This was a trial and error process where dwell time/dwell position are adjusted to achieve optimal dose coverage to High risk clinical target volume (HRCTV) (EQD2>80Gy10) with constraints to bladder D2cc (EQD2<85Gy3) and rectum D2cc (EQD2< 75 Gy3)(Viswanathan et al., 2009).” |
| Bias | 9 | Describe any efforts to address potential sources of bias  We notably tried to reduce bias by excluding suspect cases. The analysis section also explains part of our analysis was adjusted on type on context in order to reduce bias  Page 4 |
| Study size | 10 | Explain how the study size was arrived at  “Study included 150 patients diagnosed with Carcinoma cervix (Table 1), out of which 6 patients were excluded due to loss of follow-up and since they did not receive chemotherapy”  Page 15, Table 1 |
| Quantitative variables | 11 | Explain how quantitative variables were handled in the analyses. If applicable, describe which groupings were chosen and why  Page 4 and 6  “Fisher’s exact test was performed to see the association between two categorical variables. P value of less than 0.05 was considered significant.”  “Complete response was seen in patients with mean EQD_2_ of 78.67 Gy_10_, 83.33 Gy_10_, 84.23 Gy_10_, 85.63 Gy_10_ in stages I, II, III, IVA respectively (Table 2)” |
| Statistical methods | 12 | (*a*) Describe all statistical methods, including those used to control for confounding  These are described in the method section  Page 4  “The statistical analysis was carried out using the statistical tool SPSS version 22.0.  Continuous variables were presented as Mean ± Standard Deviation, Median, Min: Max, Q1:Q3. Categorical variables were presented as frequency and percentage. Fisher’s exact test was performed to see the association between two categorical variables. P value of less than 0.05 was considered significant.” |
|  |  |  |
|  |  |  |
|  |  |  |
|  |  |  |
| Results | | |
| Participants | 13* | Report numbers of individuals at each stage of study—eg numbers potentially eligible, examined for eligibility, confirmed eligible, included in the study, completing follow-up, and analysed  Page 15, Table 1  “Study included 150 patients diagnosed with Carcinoma cervix (Table 1), out of which 6 patients were excluded due to loss of follow-up and since they did not receive chemotherapy.” |
|  |  | (b) Give reasons for non-participation at each stage  Not applicable |
|  |  | (c) Consider use of a flow diagram  Not applicable |
| Descriptive data | 14* | (a) Give characteristics of study participants (eg demographic, clinical, social) and information on exposures and potential confounders  Table 1 describes the participants |
|  |  | (b) Indicate number of participants with missing data for each variable of interest  The total numbers of recorded data for each variable are stated in variable headline of each table |
| Outcome data | 15* | Report numbers of outcome events or summary measures  All numbers are reported in Tables |
| Main results | 16 | Give unadjusted estimates and, if applicable, confounder-adjusted estimates and their precision (eg, 95% confidence interval). Make clear which confounders were adjusted for and why they were included  Results section, Page 4  “The mean age was 53.63± 10.21 years. The CR observed in stage I, II, III, IVA are 60%, 79.4%, 86% and 76.2% respectively. In the ulceroproliferative group, 113 patients (81.3%) had a complete response while 1 patient (33.3%) had a complete response in the endophytic group. CR was observed in 82.7% of patients in the squamous cell variant while 64.8% of patients with adenocarcinoma variant had a complete response. P value found to be significant. P value was 0.0149, depicting that the histopathological variant of cervical cancer had a statistically significant effect on the outcome in our study, showing squamous cell carcinoma has the higher chance of clinical complete response when compared to adenocarcinoma.” |
|  |  | (*b*) Report category boundaries when continuous variables were categorized  Results section, Page 4  “79.2 % of cisplatin-treated patients and 87.5 % of carboplatin-treated patients had a complete response (Figure 2). It was found that there is no significant association between the type of chemotherapy received by the patient and clinical response at the end of three months. The p-value is 0.837, indicating that both carboplatin and cisplatin had similar outcomes and did not cause any significant alteration in the outcome.”  Figure 2, Figure 3 |
|  |  | (*c*) If relevant, consider translating estimates of relative risk into absolute risk for a meaningful time period  Result section , Page 5  “Lymph nodal status in the initial MRI was compared with the radiological response (MRI) at the end of three months. Patients without significant regional lymphadenopathy had 79.6% complete response, patients with pelvic lymphadenopathy had a 80% complete response and patients with paraaortic lymphadenopathy had a 100% complete response at the end of 3 months in the current study. Patients with pelvic lymphadenopathy also showed a higher percentage of progressive disease (12.5% versus 10.2%) when compared to patients with no significant lympadenopathy. “ |
| Other analyses | 17 | Report other analyses done—eg analyses of subgroups and interactions, and sensitivity analyses  Not applicable |
| Discussion | | |
| Key results | 18 | Summarise key results with reference to study objectives  Key results are described at the beginning of discussion section (page 7),. They also are summarized in the conclusion section ( page 13)  “The outcome of ICBT/ISBT depends on many factors - individual expertise, quality and timing of implantation, anatomy of treatment site, extent of vaginal packing, and type of source. This can lead to an excess of toxicity if the source is placed too close to a critical structure” |
| Limitations | 19 | Discuss limitations of the study, taking into account sources of potential bias or imprecision. Discuss both direction and magnitude of any potential bias  Page 13, Conclusion section  Long follow-up periods are necessary for the evaluation of response, long-term or late toxicity, and survival outcomes, which could be a limitation of our study. We also did not have an uniform pattern of scheduling between EBRT and brachytherapy in patients referred from several other centers in the city for brachytherapy at our center. |
| Interpretation | 20 | Give a cautious overall interpretation of results considering objectives, limitations, multiplicity of analyses, results from similar studies, and other relevant evidence  Page 13  References were added where possible, and discussed. Limitations were taken into account |
| Generalisability | 21 | Discuss the generalisability (external validity) of the study results  Not applicable |
| Other information | | |
| Funding | 22 | Give the source of funding and the role of the funders for the present study and, if applicable, for the original study on which the present article is based  Not applicable |

*Give information separately for exposed and unexposed groups.

**Note:** An Explanation and Elaboration article discusses each checklist item and gives methodological background and published examples of transparent reporting. The STROBE checklist is best used in conjunction with this article (freely available on the Web sites of PLoS Medicine at http://www.plosmedicine.org/, Annals of Internal Medicine at http://www.annals.org/, and Epidemiology at http://www.epidem.com/). Information on the STROBE Initiative is available at www.strobe-statement.org.
