# Supplementary material for: Clinical and dosimetric correlation in terms of treatment response, bladder and rectal toxicities in cervical cancer patients treated with cobalt 60 high dose rate brachytherapy
Source: PeerJ. 2024 Aug 22;12:e17759. doi: 10.7717/peerj.17759 (PMC11345003; doi:10.7717/peerj.17759)
Supplement: Supplemental Information 3 [file peerj-12-17759-s003.pdf]

ANNEXURE -I

PROFORMA

- Hospital no:
- Brachytherapy no :
- Age:
- Chief complaints:
- Past history : Diabetes mellitus/Systemic hypertension/tuberculosis/epilepsy
- Date of First Diagnosis(biopsy):
- Complete Pathological Diagnosis:
- Investigations for Staging:
  - ❖ MRI / CT Scan:
  - ❖ Chest X ray : Normal/Abnormal
  - ❖ Basic blood investigations: Normal/Abnormal
  - ❖ HIV Status : Positive/Negative
  - ❖ HBs Ag Status : Positive/Negative
- FIGO Stage:
- Radiotherapy details (External beam radiation)(Technique):
  - ❖ Total dose:
  - ❖ No of fractions:
  - ❖ Any nodal boost:
  - ❖ From ………………to……………………
  - ❖ Any Gap between treatment :
- Chemotherapy details:
  - ❖ Is chemotherapy given: YES or NO
  - ❖ IF YES
    - ❖ Agent used:
    - ❖ Number of cycles:
- Interval between CT-RT and Brachytherapy : days
- Brachytherapy details:

Address:

Phone no:

Scanned with OKEN Scanner

BR.NO: ________/________ **ICBT/ISBT:**

Intra-Uterine Tandem: Flange:

Ovoid : Needle/s (if any):

Simulation : CT / X-RAY / NONE

**HDR Dose Prescription :** ________ **Gy / # in** ________ **#**					**EBRT Dose** ________ **Gy in** ______ **#**	
**Description**	**Fraction Number**				**Tumor BED** (in Gy_{10})	
	**1**	**2**	**3**	**4**	**OAR BED** (in Gy_3)	
Date					**Tumor EQD2** (in Gy_{10})	
Time of treatment					**OAR EQD2** (in Gy_3)	
Dose /# (in Gy)					**Total values of BED & EQD2 Inclusive of EBRT + HDR**	
HRCTV D100 (%)					**BED to Tumor**	
HRCTV D90 (%)					**EQD2 to Tumor**(in Gy_{10})	
Dose of **Bladder** D2cc (Gy)					**EQD2 to Bladder** (in Gy_3)	
EQD2 of **Bladder** D2cc (Gy_3)						
Dose of **Rectum** D2cc (Gy)					**EQD2 to Rectum** (in Gy_3)	
EQD2 of **Rectum** D2cc (Gy_3)						
Dose of **Sigmoid** D2cc (Gy)					**EQD2 to Sigmoid**(in Gy_3)	
EQD2 of **Sigmoid** D2cc (Gy_3)						

Scanned with OKEN Scanner

Total duration of brachytherapy:

Total duration of treatment:

Follow up at 3 months:

- Clinical Response: Complete response / Partial response / progressive disease
- Acute Complications

Bladder complications: RTOG Grade:

Rectal complications: RTOG Grade:

Scanned with OKEN Scanner
